# Selected Mechanical Properties of Glue-Laminated Timber Produced from Locally Repaired Timber

**DOI:** 10.3390/ma15228112

**Published:** 2022-11-16

**Authors:** Adam Derkowski, Marcin Kuliński, Adrian Trociński, Sławomir Krzosek, Radosław Mirski

**Affiliations:** 1 Department of Mechanical Wood Technology, Poznań University of Life Sciences, ul. Wojska Polskiego 28, 60-627 Poznań, Poland; 2 Department of Mechanical Processing of Wood, Institute of Wood Sciences and Furniture, Warsaw University of Life Sciences—SGGW, 159 Nowoursynowska St., 02-776 Warsaw, Poland

**Keywords:** finger joint, beams, mechanical properties, lumber, wood defects, knots

## Abstract

This study aimed to evaluate the mechanical properties determined in a 4-point bending test of beams made of lumber from which knots had been locally removed and the resulting loss replaced with sound wood. Three sets of beams were prepared, which differed in the number of layers/lamellas and the position of the lamellas from which edge knots were removed. All the lamellas used in the tests were subjected to a modulus of elasticity assessment. In addition to the distribution of defects, it determined the position of a given piece in the beam structure. The tests showed that high mechanical properties could characterise the beams produced in this way, i.e., a modulus of elasticity close to 12 kN/mm^2^ and a strength above 40 N/mm^2^, if the lamellas without knots were located below the outer tension lamella. Significantly better results were obtained when PUR glue was used in the inserts rather than MUF. In this case, beams with an improved outer lamella in the tension zone using semi-circular inserts glued with PUR glue had an average strength of 34.6 N/mm^2^.

## 1. Introduction

The growing demand for structural timber contributes to a significant increase in production capacity, producing laminated timber. Despite the considerable disadvantages of technologies making glulam (laminated beams—also known as glulam timber), mainly related to labour intensity and the need for specialised machinery, it should gradually compete with solid wood in building structures. The sourcing of essential components such as roof structures, rafters, or purlins is generally not problematic, even for small and poorly machined sawmills. However, in addition to the primary elements, which are usually oversized anyway, this processing produces sawn timber, which is more challenging to use for construction purposes. Glulam has better mechanical properties than solid wood [1] and allows for the better use of roundwood harvested from the forest. The basic requirements for GL (glue-laminated timber) beams are well described for softwood and poplar species in EN 14080: 2013 [2]. However, this does not mean an end to research into this material. This research is generally concerned with several aspects, i.e., assessing the quality of beams made of hardwood species [3,4,5,6,7,8,9], the introduction of new species from the tropics [10,11,12,13,14,15], FEM numerical modelling [16,17,18,19,20], and load-carrying capacity improvement, most often by strengthening the beam in the tension zone [21,22,23,24,25]. Continental pine wood, in general, is troublesome for the production of structural components because it has numerous knots, occupying up to 50–70% of the surface area of a given piece of lumber. Wood, especially pine, with knots in the tension zone, regardless of the type of knot (healthy or loose), loses its ability to transmit stress [26]. However, it is not only knots that reduce the quality of structural lumber but also fibre twists or severe cracking [27,28,29,30]. Removing these defects and reconnecting fragments already without defects using the finger joint is now a widespread and known method [31,32,33,34]. However, removing the previously mentioned defects results in losing a considerable volume of raw material. The greater the raw material, the larger the cross-section of the ‘repaired’ piece. For these reasons, attempts are made to locally reinforce timber or structural beams [35,36,37]. The cases described mainly relate to the local reinforcement of the weakened area by gluing high-modulus materials. The local or global reinforcement of structural lumber through various types of strapping, steel, or polymer bars [38,39,40,41,42,43] has not found widespread use. In the WoS database, more than 450 papers related to timber reinforcement can be found. Only a few of the solutions have been applied in industrial practice. This is probably due to the labour-intensive nature of the processes involved in bonding these links, their unit cost, and the generally relatively low effect of the increase in load-bearing capacity on the overall process cost. These methods are much more successful for the renovation of structural beams [44,45]. Our previous work shows [46] that pine wood knots occur every 40–60 cm in such a grouping, or individually, that the most common length of the removed part is between 8 cm and 12 cm. This results in a material loss of 30%. For these reasons, glulam lumber is often produced from spruce, in which relatively few knots are relevant to Glulam strength. In our experience, 12-metre GL beams made from lumber may have as few as four finger joints along the length of the lamellas. It is, therefore, not excluded that one of these has been created by the need to join to length. In the case of pine lumber, knotless pieces are rare. In addition, even if the lamellas have a high density and linear modulus, they must not even have small edge knots if they are to be used as a face layer in the tension zone.

For this reason, methods are being searched for which, on the one hand, are easy to implement and, on the other, make maximum use of the available raw material.

The primary aim of our research was to increase the proportion of pine lumber in the production of GL beams. Hence, we only removed edge knots locally and filled this area with defect-free wood. With easy access to machining centres, this type of solution seems to be applicable. Therefore, this study aimed to evaluate the mechanical properties determined in a 4-point bending test of beams made of lumber from which knots had been locally removed and the resulting loss replaced with healthy wood.

## 2. Materials and Methods

The research was carried out in two stages. In the first stage, the effect of edge knots and the quality of the lumber after replacing the knots with healthy wood was determined. For this purpose, five sets of pine lumber were prepared with the following dimensions: lengths of 85 cm and 91 cm, widths of 100 mm and 140 mm, and a thickness of 40 mm. The average density of the lumber samples used in the study was 575 kg/m^3^ (at a moisture content of 8.92%). The average density is, therefore, close to the average value obtained for the same lumber; however, it was not cut into smaller samples and had dimensions of 140 × 40 × 3500 mm [46]. The standard deviation was as high as 81 kg/m^3^, indicating that the coefficient of variation takes on a relatively high value of over 14%. Thus, in terms of density, the prepared test material corresponds to the lumber used to manufacture the beams.

**Figure 1:**
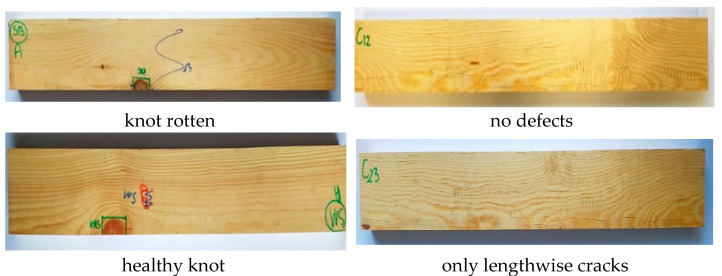
Surface appearance of lumber without and with defects.

**Figure 2:**
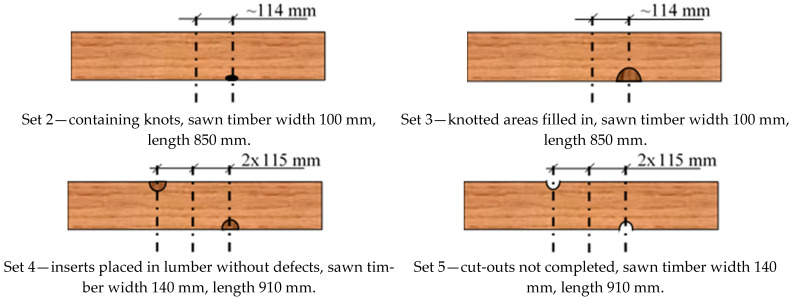
Schematics for the analysed sets.

**Figure 3:**
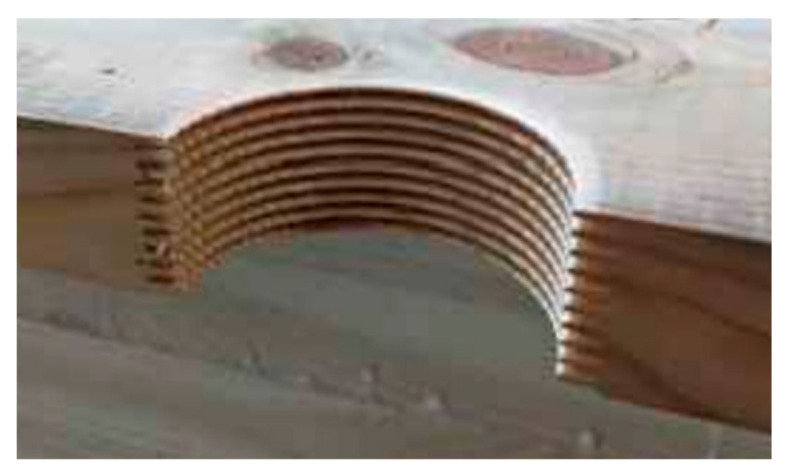
Appearance of the socket and the insert used to fill in the space left by an edge knot.

The first set was lumber with no significant edge defects, the second set was lumber with edge defects, and the third set was lumber with edge knots that had been replaced with a semi-circular insert after cutting—the joint was made with the finger joint; in the fourth set, two inserts were glued in on opposite sides; and in the fifth set, the lumber was examined without filling in the area after cutting out the defect. Unfortunately, in the first two sets, the defects were not precisely in the same place in each plank—the test specimen. The average distance from the centre of the sample length was 11.4 cm. Examples of the selected lumber are shown in Figure 1, while diagrams for sets 2–5 are shown in Figure 2. Each set consisted of a maximum of 12 specimens. Defects were removed with a 20 mm fi shank cutter, using a Homag machining centre which cut out an area (half-circle) with a 30 mm or 40 mm radius. Then, in the case of the finger insert, this area was milled with a milling cutter. The appearance of the socket and insert is shown in Figure 3. The inserts were glued in using a Rakoll GXL-4, D4 mounting adhesive.

**Figure 4:**
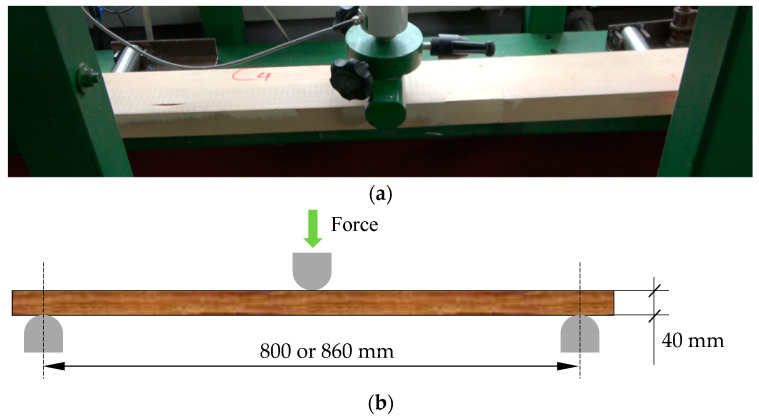
(**a**) Arrangement of the specimen in the testing machine, (**b**) loading diagram.

The specimens thus prepared were subjected to static bending strength testing in a 3-point bending scheme. The strength tests also determined the linear flexural modulus. For sets 1–3, the support spacing was 800 mm. While for sets 4 and 5, a spacing of 860 mm was adopted due to the larger width of the specimens. The specimen layout in the test machine and loading diagram are shown in Figure 4. The static bending strength (*f_m_*) was determined on the basis of the maximum force and the smallest cross-section.

A test machine capable of applying a maximum force of 50 kN was used (SAM 50 kN, Poznan, Poland). The crosshead speed was 15 mm/min, which resulted in damage to the specimen between 90 s and 120 s.

In the second stage, sets of beams were prepared using lumber from which the edge knots had been removed. The lumber used in the study was the so-called main pine lumber, which was 40 mm thick, 138 mm wide, and 350 cm long. Before the removal of the selected defects, the lumber was subjected to an elastic modulus evaluation in a 4-point bending test. The lumber was laid flat as described earlier [47], assuming a force arm of 1 m. The distance between supports was also 1 m. Based on the knowledge of the modulus of elasticity, sets were prepared as follows: lumber with a higher modulus of elasticity was further away from the centre axis of the beam, and lumber with a lower value was closer to the centre of the beam. The beams were formed as 6 or 8 layers. 

Two types of beams were manufactured containing lumber from which edge knots had been removed:

- Eight-layer beams, designated as SW; for these beams, all edge knots were removed from the lamellas below the outer tension lamella (Lam. 2 ÷ Lam. 8). The outer tension lamella consisted of lumber with a high rope modulus (>15 GPa) with no significant defects on its surfaces;
- Eight-layer beams, designated as P2, for which the outer tension lamella was made of lumber with a high modulus of elasticity (>15 GPa) and had no significant defects on its surfaces;
- Six-layer beams, designated as T, for which edge knots were removed from the outer lamella only.

After the defect was cut out, the inserts were glued in with melamine-urea-formaldehyde glue with 50% hardener. The same glue was used when bonding the beams. In this case, the designation was extended with the letter M. In the second case, PUR D4 polyurethane glue from Anser was used. The beams produced in this way were marked with an additional letter U.

**Figure 5:**
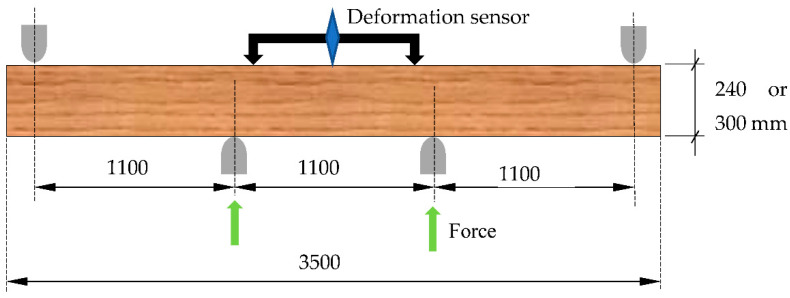
Schematic of the tested beam.

Immediately before pressing the prepared sets, each piece of lumber was planned, and its final thickness was 1 to 1.5 mm less than the initial thickness. The beams were manufactured to be cold in the press, using the MUF 1247 adhesive at 220–240 g/m^2^ mixed with 2526 hardener at 10% of the dry weight of the resin. Both products were from Akzo Nobel. The mixture was prepared to take into account the conditions in the laboratory hall. The adhesive was applied using a roller applicator. After loading the press, a pressure of 0.5 ÷ 0.55 MPa was applied. A minimum of 5 beams were pressed for each variant. Pressing was carried out on an industrial press equipped with hydraulic cylinders designed to produce glued structural elements (FOST, Czersk, Poland). The beams stayed in the press for a minimum of 4 h due to the assumed amount of hardener added to the resin. After a conditioning period of about two weeks, the beams were subjected to flexural strength and modulus of elasticity in a 4-pound bending test. The beams were air-conditioned in the laboratory hall. Laboratory conditions were controlled: the temperature was 21 ± 2 °C and the humidity was 55 ÷ 65%. A testing machine capable of exerting a maximum force of 500 kN (SAM 500 kN, Poznan, Poland) was used to assess the bending strength and modulus of elasticity. A diagram of the measuring system is shown in Figure 5. The machine is equipped with a hydraulic drive. The beams were loaded at a speed of 0.20–0.25 mm/s. Before assessing the mechanical properties, the moisture content of each beam was tested. The moisture content was determined using a HIT-3 resistance moisture metre. The modulus of elasticity (Em) was determined by loading the beam eight times with a force (F) of about 25 kN, but the strain was only recorded for the last five tests. The strain was measured by strain sensor MSL 50.102 PA (Larm, Czech Republic). The bending strength (*f_m_*) was determined using the basic four-point bending equation.

After a period of air conditioning (T = 22 ± 2 °C, RH = 65 ± 5%), the beams were tested for their bending strength (*f_m_*) and the modulus of elasticity (in the four-point bending test. The obtained results were analysed statistically and compared with the results of previous studies. Statistica software version 13.0 (Version 13.0, StatSoft Inc., Tulsa, OK, USA) was used to carry out the statistical analysis.

## 3. Results and Discussion

**Figure 6:**
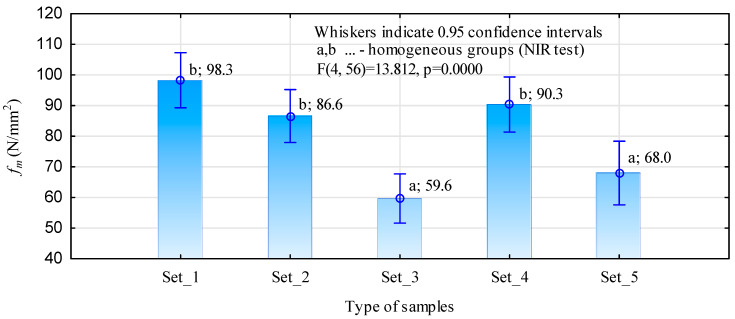
Static bending strength of the specimens prepared in the first stage of testing.

From the data shown in Figure 6, it can be seen that removing the edge knots or only gluing in the semi-circular inserts resulted in static flexural strengths similar (no statistical differences) to those of the lumber without edge knots. A conservative test (NIR) was used for the statistical analysis, specifically the determination of homogeneous groups; despite this, the research shows similarity in the mean strength values for the Set_1, Set_2, and Set_4 samples. The observed differences for these samples are around 10%. The advantage of the specimens with inserts is probably influenced by the insertion point’s remoteness from the specimen’s centre. The insert is not directly under tension, which results in a lower bending moment acting on it.

**Figure 7:**
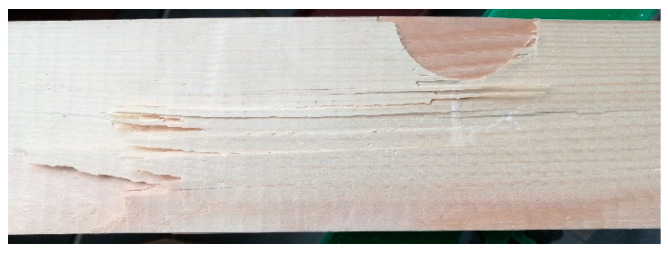
Example of a damaged specimen containing a semi-circular insertion.

In Figure 7, it can be seen that damage occurs directly under thrust. However, the specimens containing edge knots show the lowest static bending strength of almost 30% compared to the system where the knots were replaced by inserts (Set 3/Set_2). 

Although the number of specimens is small (12 per set), the decrease in strength of the specimens without inserts is not only the result of a reduction in the cross-section of the analysed samples. By cutting out part of the timber, the fibres are cut. There is some disruption of the structure of the lumber in this section, and there is a notch-like effect. By gluing the material in this area using the finger joint, some improvement in strength is achieved (Set_4 = 90.3 N/mm2 against Set_5 = 68 N/mm^2^). However, despite the demonstrated lack of differences, it must be assumed that the effect is also dependent on the wood’s structure (grain arrangement) at the defect repair site. Additionally, ultimately, one should not count on achieving a load-bearing capacity similar to that of wood without defects. The test results described above, due to the small number of tests carried out, should be regarded as an indication of how individual batches of lumber may behave rather than an attempt to indicate specific values for static bending strength. 

When preparing the lumber sets for the manufacturing of the beams, as mentioned in the methodology for the first assumption, lumber with a high modulus of elasticity and no edge defects was selected for the outer layer. A sorter assessed each piece. Table 1 shows the average moduli of elasticity of the lamellas used in the study, arranged in the different layers of the beams manufactured. Lam. 1 is the outer lamella of the tension zone. As seen from the data in Table 1, the arrangement of the layers on the tensile side in the designed beams is similar. Nevertheless, the best strength effects are expected from the SW_M set and the lowest from the P2 set. However, this arrangement of layers should allow for strengths above 30 MPa in each set. 

**Figure 8:**
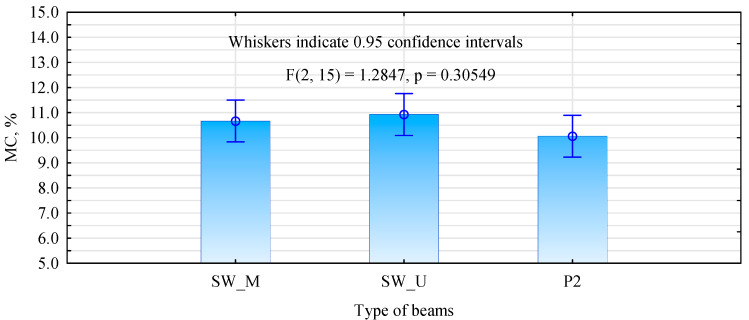
Moisture content (MC) of the beams during the bending test evaluation.

As can be seen from the data in Figure 8, the moisture content of the beams, despite having been manufactured and tested at different times, shows similar values, ranging from 10.1% to 10.9%. Therefore, the results, especially the modulus of elasticity, can be compared between the different sets of beams without any problems.

**Figure 9:**
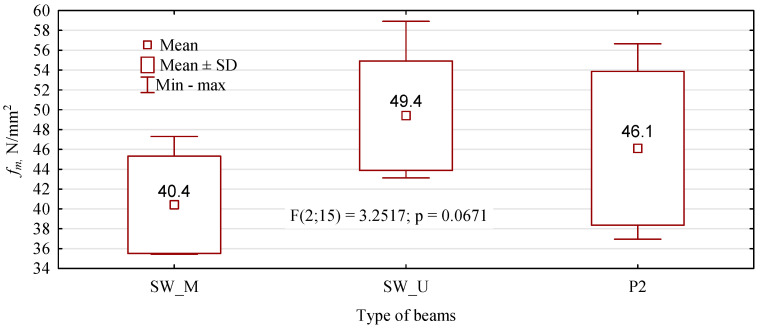
Static bending strength of beams with high-quality external tension lamella.

The obtained static bending strength values were also subjected to ANOVA analysis. The test results indicate no statistically significant differences in this property in the fabricated beams (Figure 9).

**Figure 10:**
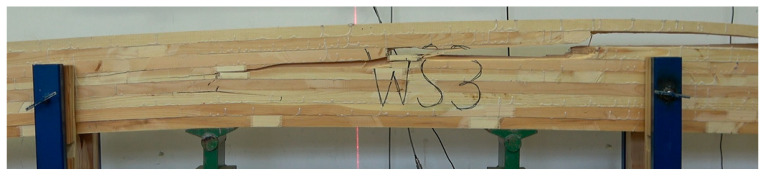
Damage image of beam type WS_U, *f_m_* = 46.7 MPa.

**Figure 11:**
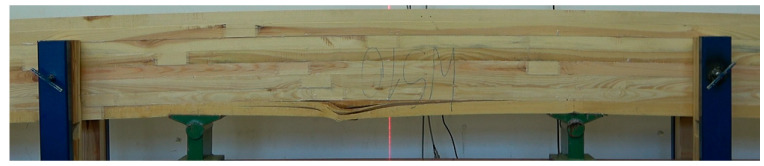
Damage image of beam type WS_M, *f_m_* = 45.2 MPa.

All beams achieved a high average strength value, exceeding 40 N/mm^2^. Additionally, it is noteworthy that the minimum value is above 34 N/mm^2^. It is, therefore, in line with expectations. However, the expected effect was not achieved, as the SW_M beams had the lowest average static bending strength value, despite the best quality outer layer. In addition, beams with inserts did not show significantly higher values than those without them. Instead, a reduction in internal distribution was achieved. The minimum and maximum values are much closer to the mean value. The effect obtained cannot be clearly attributed to the inserts introduced. This is because the inserts were only placed below the outer tension lamella. The outer lamella carries almost 58% of the applied load, while the lamella below carries only just under 30%. However, the influence of the glue used to paste the inserts seems to become apparent. The strength of beams where PUR glue was used is more than 22% higher than beams with inserts glued in with MUF glue. We have two cases to analyse the causes of the loss of load-bearing capacity of manufactured beams in detail. In the first, the damage is propagated by inserts glued in with PUR glue (Figure 10), but importantly, the outer layer is not damaged. In the second case, the loss of load-bearing capacity is caused by damage to the outer compression lamella (Figure 11). Both types of beams, however, show similar strength.

**Figure 12:**
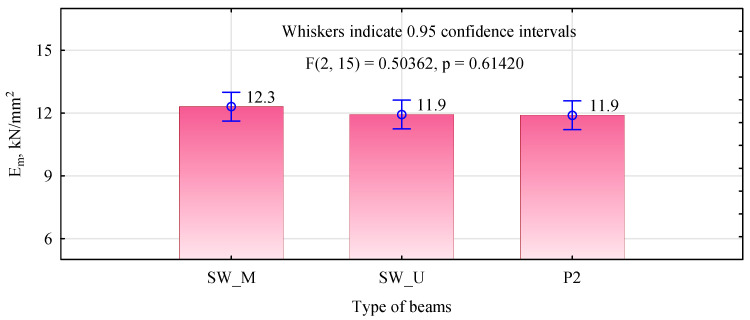
Analysis of the elasticity modulus ANOVA of manufactured 8-layer beams.

The modulus of elasticity values obtained are slightly lower than expected based on the knowledge of the elastic characteristics of the lumber used in the tests (Figure 12). Approximately 15 percent higher values of the modulus of elasticity were expected. However, the general trend was maintained, with the SW_M type beams having the highest elasticity and the other two variants having similar elasticity. 

It was decided to not directly compare 8-layer beams with 6-layer beams, primarily because the same bending scheme was assumed. In the case of 6-layer beams, this results in beams bending close to the normative support spacing (normative = 18 × h ± 3; assumed = 14.5 × h). For 8-layer beams, this spacing is reduced to as much as 11 × h.

**Figure 13:**
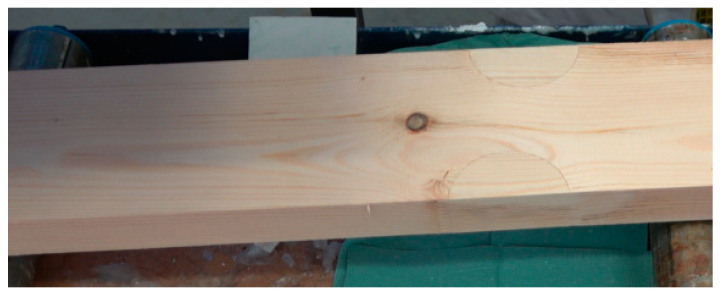
Appearance of lumber with inserts intended for the outer tensile layer.

Table 2 shows the average values of the modulus of elasticity of the sawn timber used in the tests, broken down by the location of the individual lamellas in the beam section. In this case, the outer layers consisted of lumber with significantly lower moduli of elasticity than in the case of 8-layer beams. The moduli of elasticity of the outer lamellas corresponded more to the values of the lamellas positioned second below the tension zone. The two sets appeared to be similar in terms of the quality of the lumber used. All edge knots were removed from the outer tension lamella. This could have resulted in the inserts being located opposite each other (Figure 13).

**Figure 14:**
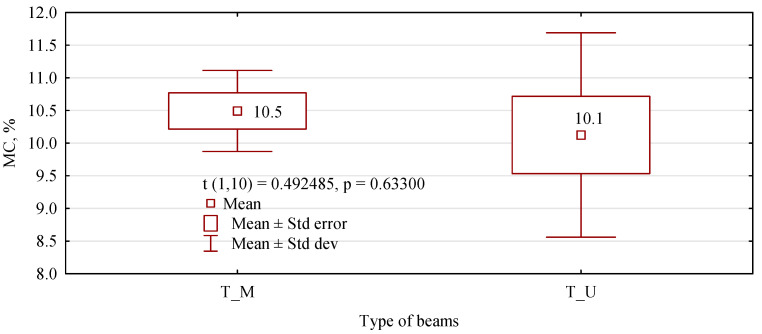
Average moisture content of 6-layer beams determined during the determination of mechanical properties in the bending test. The *t*-test is a statistical hypothesis test in which the test statistic follows a Student’s t-distribution under the null hypothesis.

Additionally, in this sample, the analysed beams had an average moisture content at a similar level (Figure 14). However, the T_U beams showed more variability in this range.

**Figure 15:**
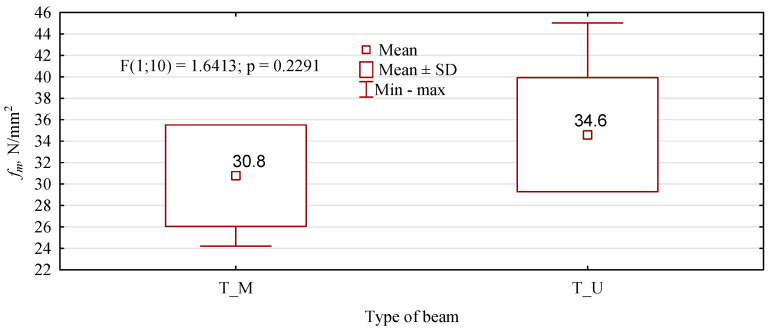
Static bending strength of beams with a modified outer layer.

The manufactured 6-layer beams showed significantly lower flexural strength values than the 8-layer beams (Figure 15). The introduction of lumber in the tension zone in which the edge knots were replaced with inserts from healthy pine timber should result in an increase in the load-bearing capacity, relative to the non-defective lumber. The theoretical bending strength, determined by the elastic moduli of the individual lamellas, should be at least 24 N/mm^2^. As the number of tests performed is very small, it is perhaps challenging to analyse this in detail. However, given the testing capacity, the number of tests performed rarely approaches 40. Very often, it does not exceed 15. In this situation, it must be assumed that the characteristic value of the bending strength will always be the smallest result. In the variants analysed, this is just over 24 N/mm^2^ for T_M beams and as much as 30 N/mm^2^ for T_U beams. Although the statistical analysis shows no basis for rejecting the null hypothesis that the mean static bending strengths are equal, the T_U beams show higher strengths than the T_M beams. MUF glue is advantageous because it is the same glue used to glue the individual lamellas together. However, the observed poorer performance with it may have a different basis.

On the one hand, the curing time is corrected by the amount of hardener, which means that gelled glue may be used. On the other hand, its higher viscosity makes it much more difficult to apply. However, these are problems of manual application of the adhesive that will not be relevant during such beams’ eventual machine manufacturing process.

**Figure 16:**
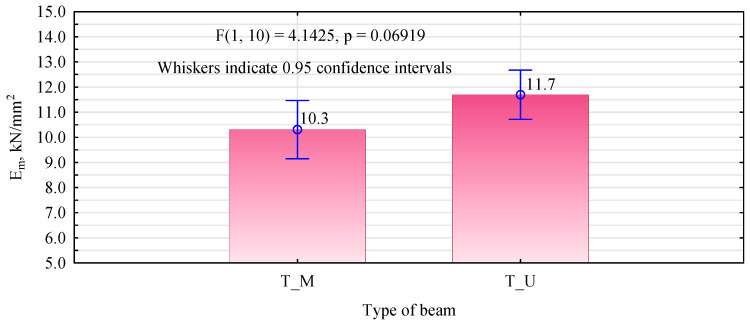
Statistical analysis of the modulus of elasticity of the manufactured 6-layer beams.

Very importantly, however, both types of beams achieved similar average static bending strength values (with a similar layer arrangement) to the G_so_ beams (described in Derkowski et al. 2022) [47]. The G_so_ beams were manufactured as 6-layer beams, with all defects removed from the outer layer, especially knots from both the edge and middle zones. The defects were removed traditionally by cutting out the entire area and joining the resulting parts using a finger joint. In addition, the lowest strength values obtained for G_so_ beams are close to those specified for T_M beams but significantly lower than for T_U beams. This means that the proposed method makes it possible to save up to 20% of the processed mass, as the removed fragments can be replaced by fragments (inserts) obtained from so-called piece waste. Of course, the insertion system and the inserts’ size need to be developed further. A readily available general-purpose cutter was used in the study. Therefore, consideration should be given to making a special tool with characteristics similar to a milling cutter designed for structural finger joints in wood. This is also supported by the fact that, even in the case of the first stage of testing, a strength equal to that of non-defective wood was not obtained. Additionally, the lower values of the modulus of elasticity determined for type T_M beams compared to type T_U beams indicate that the quality of the insert bonding is essential in the later evaluation of the mechanical properties of the manufactured beams (Figure 16).

## 4. Summary and Conclusions

The implementation of pine lumber as an essential raw material for producing glue-laminated sawn timber is an extensively studied issue in the Central European area. It is the primary forest-forming species in this area and is not widely used despite its numerous advantages. Numerous knots, which often fall out and are large, raise concerns among producers and customers about the load-bearing capacity of pieces made from such lumber. The simplest way to remedy this is to remove the questionable areas which involve a significant material loss. The solution analysed here is to remove the edge knots, which have the most significant impact on the strength of the elements in the bending test, and to replace these areas with an insert of sound wood. This solution should reduce material loss to some extent and ensure an adequate load-bearing capacity. 

The following conclusions can be drawn based on the obtained results:

- The proposed method of removing edge defects in timber makes it possible to produce structural beams with satisfactory characteristics, as determined by the 4-point bending test;
- Significantly better results were obtained when the lumber containing the semi-circular inserts was below the outer tension lamella. This is probably because the outer lamella masks the quality of the glued-in inserts;
- Slightly better results were obtained with PUR glue for gluing the inserts than with MUF resin, which is used for the manufacture of glue-laminated timber;
- Although not directly stated, the characteristics of the milling cutter making the finger joint should be similar to those of the milling cutters used to make joints for structural elements.

## Figures and Tables

**Table 1:** Average moduli of elasticity of the lamellas used in the tests for 8-layer beams.

Type	Feature	Lam. 1	Lam. 2	Lam. 3	Lam. 4	Lam. 5	Lam. 6	Lam. 7	Lam. 8
SW_M (6 pcs.)	E_m_, GPa	15.65	13.12	11.68	10.76	10.80	11.64	13.06	14.13
	SD, GPa	0.20	0.36	0.20	0.32	0.31	0.17	0.35	0.69
SW_U (6 pcs.)	E_m_, GPa	15.38	12.60	11.92	10.31	10.37	11.91	12.55	13.66
	SD, GPa	0.26	0.42	0.23	0.37	0.43	0.24	0.42	0.17
P2 (6 pcs.)	E_m_, GPa	15.25	11.60	8.73	7.92	7.92	8.92	11.67	15.30
	SD, GPa	0.22	0.40	0.15	0.43	0.54	0.46	0.40	0.26

SW/P2—facing layer of high-quality lumber, M—MUF glue, U—polyurethane glue.

**Table 2:** Average moduli of elasticity of the lamellas used in the tests for 6-layer beams.

Type	Feature	Lam. 1	Lam. 2	Lam. 3	Lam. 4	Lam. 5	Lam. 6
T_M	E_m_, GPa	12.86	11.85	10.21	10.24	11.81	12.81
	SD, GPa	0.34	0.17	0.25	0.24	0.16	0.33
T_U	E_m_, GPa	12.34	11.52	10.58	10.63	11.45	12.30
	SD, GPa	0.20	0.20	0.25	0.24	0.24	0.15

T—edge-defect-free facing layer, M—MUF adhesive, U—polyurethane adhesive.

## Data Availability

The data presented in this study are available on request from the corresponding author.
